# Mechanical Performance of Glass/Epoxy Composites Loaded with Silane-Treated Aluminum Hydroxide Fillers

**DOI:** 10.3390/polym15173514

**Published:** 2023-08-23

**Authors:** Khubab Shaker, Muhammad Adnan, Yasir Nawab, Muhammad Umair, Madeha Jabbar, Amna Siddique, Ahsan Ahmad

**Affiliations:** 1 Department of Materials, School of Engineering and Technology, National Textile University, Faisalabad 37610, Pakistan; 2 National Center for Composite Materials, School of Engineering and Technology, National Textile University, Faisalabad 37610, Pakistanynawab@ntu.edu.pk (Y.N.);; 3 Department of Textile Engineering, School of Engineering and Technology, National Textile University, Faisalabad 37610, Pakistan; 4 Department of Textile Technology, School of Engineering and Technology, National Textile University, Faisalabad 37610, Pakistan

**Keywords:** tensile, flexural, short beam shear, glass/epoxy composites, flame retardancy, silane coupling agent, Al (OH)_3_ fillers, luggage bag

## Abstract

This study investigates the influence of silane-treated aluminum hydroxide on the mechanical performance of flame-retardant composites. These composites have potential applications for luggage bags, as a replacement for conventional plastics, offering more durability and lighter weight. Glass fabric was used as the reinforcement, while epoxy was used as the matrix material. To impart flame retardancy, aluminum hydroxide nanoparticles were used as fillers in different weight % age (5%, 10% and 15%). As these are inorganic particles and have compatibility issues with the matrix material, silane-coupling agents (Dynasylan^®^ 6490 and Dynasylan Glymo) were used to treat these filler particles. Both the silane-coupling agents fraction used for treatment and the fillers fraction added to the composites were varied to determine the most optimum combination. The mechanical properties of the developed composites such as tensile, flexural, and short beam shear strength were investigated. The best results were exhibited by 10% aluminum hydroxide fillers treated with 1% (by weight) coupling agent (Dynasylan Glymo).

## 1. Introduction

The significant increase in the demand of composite materials over traditional materials is due to various attributes such as their excellent mechanical properties, design flexibility and weathering resistance [1]. The composite materials with diverse performance and functional properties are finding applications in structural, civil, aerospace, automotive, biomedical, maritime, and many other industries [1,2,3,4,5]. There is a rapid increase in the market for composite materials owing to their compatibility in various industrial and structural applications. Fiber-reinforced composites offer multiple benefits of high specific stiffness, high specific strength, durability, and damage tolerance as compared to metallic counterparts [6]. The properties of these composites are governed by their constituent elements and production methods. As the fiber-reinforced polymer composites are composed of a fiber and matrix, so their mechanical performance is governed by the chemical stability, modulus and strength of the matrix and fiber [1]. At present, glass fibers are mainly used as reinforcement in composites due to their exclusive properties such as the high temperature requirement for thermal decomposition, good electric insulation, low cost, density, chemical stability and high mechanical performance. Glass fibers’ commercialization is quite established. Glass fiber-reinforced epoxy composites reveal numerous benefits including high tensile strength and cost effectiveness [7]. Thermosetting epoxy resins have been widely used with glass fibers in household applications and as structural elements in several industries such as defense, automobile, marine, aerospace, and so on. These widespread applications are due to their superior mechanical strength and some other unique properties of epoxy resins such as heat resistance, chemical resistance and excellent adhesion. However, their poor flame retardancy is a main drawback that limits their uses in such applications [8,9,10]. They are easily flammable due to the chemical characteristics of epoxy thermosets [11]. Various multistage chemical and physical changes occur during the burning of epoxy composites. A redox reaction results in combustion, which liberates heat into the atmosphere. Combustion occurs as a result of redox reactions which release heat that has no time to liberate into the environment. Flame retardancy is an important property for composite materials, especially when used for structural or household applications. To overcome this setback of glass fiber-reinforced epoxy composites, flame retardants (FRs) are used as a necessary additive [12], being the convenient and cost-effective way to introduce flame retardancy. The various functional aspects of materials are achieved by the addition of fillers or some functional treatments. Some common advantages of the addition of fillers in composites include an improvement in the properties and a cost reduction [13]. 

The burning of any substance is governed by three aspects including a heat source, oxygen availability, and fuel presence [14]. Hence, a flame-retardant material should have the provision to deal with these three aspects. The mechanism of flame retardants includes endothermic degradation, thermal shielding, gas-phase radical quenching and dilution of the gas phase. The most commonly used filler particles for thermal stability are halogenated flame retardants (FR) (containing chlorine or bromine bonded to carbon) and organophosphorous flame retardants (ammonium polyphosphate [15], aluminum phosphate [16], zirconium phosphate [17], etc.). Halogenated flame retardants have considerable toxicity, i.e., either they degrade into toxic compounds or the degradation products are primary toxic agents [18]. The entire class of organo-halogen flame retardants is suggested to be potentially harmful. Hence, due to regulatory actions, their use is decreasing [19]. A recently published study [20] explains the effect of phosphorus and chlorine plasticizers on the fire safety and strength of composites. A modified epoxy polymer by introducing a plasticizer presented improved fire protection as the limiting oxygen index (LOI) increased from 25 to 31% by volume and the mass loss on ignition in air decreased from 2 to 9%. They also reported a significant increase in the mechanical properties of modified epoxy composites: the tensile strength increased by 38–46%, impact strength resistance increased by 2–4-fold, and the bending stress resistance increased by 25–32%.

The aluminum hydroxide, Al (OH)_3_, is a commonly used cost-effective inorganic flame retardant that is non-toxic in nature. It plays the role of a flame retardant and a filler as well. In aluminum hydroxide, aluminum ions form ionic bonds with hydroxide ions. These ionic bonds’ breakdown starts at a temperature of 220 °C. When the temperature goes above 220 °C on heating, it starts liberating water molecules. Hence, the liberated water molecules provide natural flame retardancy to aluminum hydroxide fillers. It has been explored in a variety of compounds such as rubbers, foams, latex, wires, etc. These flame retardants are non-halogenated and produce low smoke and fumes [21].

Aluminum phosphate (APP) is a fire-retardant form of phosphorus that has had its ability in the polymer nanocomposite (polystyrene, polypropylene, and polyvinyl alcohol) recently explored. A fascinating area to study in terms of mechanical and flame-retardant potential is the application of aluminum phosphate in a polyester framework. The application of 5 wt.% aluminum phosphate provides optimal fire-retardant and mechanical properties of modern-reinforced fiberglass fire retardant. This modern fire-retardant blend has presented a stronger relationship between polyester and fiberglass and aluminum phosphate, which reflects its mechanical and flammability properties [16].

Ammonium polyphosphate is environmentally sensitive, fire resistant, and also commonly used for increasing polymers’ resistance to fire. The Dia-APP (diatomite/ammonium polyphosphate) TPP (triphenyl phosphate) double-layer microencapsulated structure was effectively formulated and is commonly used as a fire retardant in an unsaturated polyester resin (UPR) matrix. The Dia-APP-TPP thermal behavior revealed that Dia, APP, and TPP had synergistic effects in char formation. UPR composites’ LOI value rose from 19.2% to 27.8% with 30 wt.% integration [15].

Luggage bags are widely used, and their application areas vary from high-end to daily use (travel luggage or business luggage) and fashion products. Nearly half (45%) of the luggage bag industry consists of travel bags, which are mostly made of fabric, metals or plastics. These materials have higher weight, low mechanical performance and are flammable. The considerable development in the tourism sector has fostered the demand for travel bags. The composite materials developed in this study may be used as an alternative material for luggage bags, overcoming these issues. An important issue while using flame retardants is that generally, the incorporation of flame-retardant fillers in polymeric composites causes a reduction in its mechanical performance due to poor interfacial adhesion [18,22]. However, it can overcome by treating the flame-retardant fillers with silane-coupling agents for improved interfacial adhesion and ultimately enhanced mechanical properties [23]. In recent years, several methods have been adopted to improve the mechanical properties of epoxy-based nanocomposites by using nano-fillers including nano-silica, carbon nano-tubes, nano-clay and nano-alumina [24]. The properties of the composite material change depending upon the type and amount of filler used. The addition of CaSO_4_ filler particles in glass/epoxy composites resulted in an increase in the hardness and tensile strength of the composites [25]. The influence of mica particles as fillers on the mechanical properties of unidirectional glass fiber epoxy composites was discussed in [26]. Apart from strengthening the tribological properties of composites, mica particles also improved the compression strength and hardness of the materials. Silane-coupling agents have been used to modify the mechanical properties of glass fiber-reinforced polybutylene terephthalate composites. A significant improvement in the tensile properties of the resultant composites is reported [27]. Glass fiber contains hydroxyl groups on its surface. Therefore, the most ideal way to improve the dispersion and interface bonding between the polymer and fiber is the modification with silane coupling agent.

A group of researchers [28] used silane-coupling agents to improve the interfacial adhesion and mechanical properties of glass fiber-reinforced polybutylene terephthalate composites. In another study [29], three different silane-coupling agents have been used to improve the mechanical properties of basalt fiber-reinforced poly (butylene terephthalate) composites. The following coupling agents—namely, propyl methacrylate, trimethoxy silane and triethoxy silane—were used. They reported a slight increase in flexural strength and a significant increase in flexural modulus and tensile strength. Aminopropyl tri-methoxy-silane has also been used for the surface modification of graphene nanoplatelets with the aim to increase the tribological and mechanical properties of epoxy composites [24]. The effect of saline modification on the wear behavior and mechanical properties of epoxy-based nanocomposites has been explored by using silane-modified graphene in various contents. Compared to an unmodified graphene nanocomposite, the silane-modified graphene epoxy nanocomposite presented improved wear behavior, and mechanical properties such as the interlaminar shear, compressive and tensile strength also increased [24]. In [30], the effect of incorporation of a phosphorous-based coupling agent on the flame retardancy and mechanical properties of glass fiber poly butylene terephthalate composites has been studied. A significant improvement in flame retardance as well as tensile properties by the use of a novel synthesized phosphorus-based coupling agent was reported. 

This study aimed to develop a novel glass fiber-reinforced (FR) polymeric composite luggage bag with improved mechanical performance. The lightweight luggage bags will also contribute toward a better economy and less carbon emissions in the environment. 

## 2. Experimental

### 2.1. Materials

The materials used for this study include reinforcement, a matrix, aluminum hydroxide fillers and silane-coupling agents. The specifications of these materials are given in Table 1.

### 2.2. Methodology

**Figure 1:**
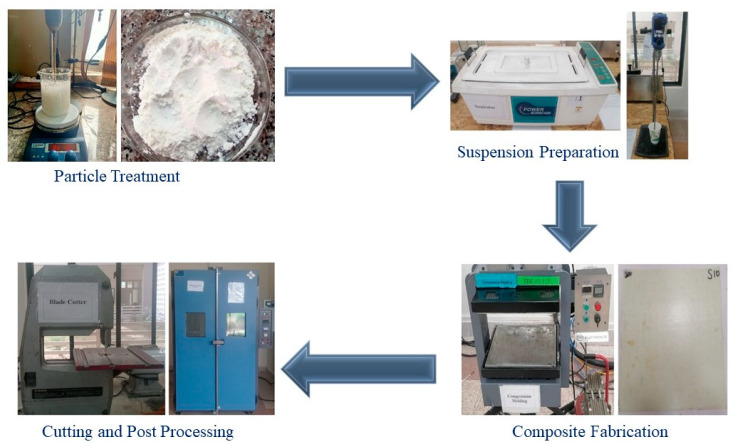
Methodology adopted for current study showing images of different stages.

The methodology adopted for this study comprises five distinct stages including the treatment of aluminum hydroxide fillers with silane-coupling agents, the dispersion of filler particles in resin to obtain a stable suspension, composite fabrication using this suspension as a matrix and glass fabric as reinforcement, composite post-processing and testing. The schematic for treatment, dispersion and composite development is given in Figure 1.

At the first stage, aluminum hydroxide Al (OH)_3_ nanoparticles were dispersed in ethanol by means of high shear mixing. A silane-coupling agent (by 1 wt.%) was added drop wise to it, and stirring was continued for 30 min. Afterwards, the solution was placed in an oven for drying purpose at 70 °C temperature. The powder obtained after drying was dispersed in the epoxy resin using a sonication bath. To obtain a homogeneous suspension, sonication was performed for 30 min at room temperature, and a hardener was added to it. After thorough mixing, this suspension was uniformly applied on glass fabric plies. Cross-ply (0°/90°) composites were prepared using 8 plies of glass fabric reinforcement and epoxy resin. The woven glass fabric of areal density 198 ± 2 g/m^2^, thread density 20 ends/inch and 20 picks/inch was used in this study. The details of the composite samples fabricated during this study are given in Table 2. 

The hand-lay-up method was used for the fabrication of composites. Glass fabric was cut into plies of 260 × 260 mm size. The fabric plies were manually placed on the mold surface in dry form and were impregnated with epoxy resin suspension with the help of a brush. The resin impregnation process was completed by using a flat bar for the even distribution of resin because it squeezes air out of the fabric. Each layer of glass fabric was firmly placed over the previous layer in a proposed stacking order to ensure there were no air pockets between plies. Finally, the composite panels were obtained using a compression molding technique and a hydraulic compression molding press by keeping the clamping force at 2.0 Ton, and the temperature for both the upper and lower platen was 120 °C for 30 min. The prepared composites were post-cured at 120 °C for about 3 h using a drying oven. Composite panels were cut by a vertical saw cutter according to the required dimensions of different tests. Cut samples were additionally scrubbed with the use of a laminate polishing machine for a smoother finish at the edges.

### 2.3. Testing

**Figure 2:**
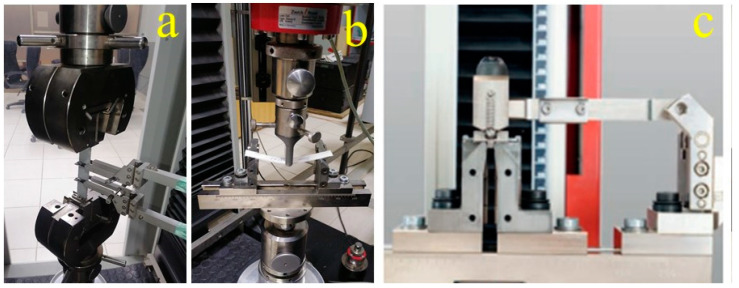
Testing equipment: (**a**) Tensile tester; (**b**) Flexural tester; (**c**) Short beam shear strength tester.

The list of testing equipment with their ASTM standard for the developed composite is given below in Table 3, and equipment images are shown in Figure 2.

## 3. Results and Discussion

### 3.1. Tensile Properties

The tensile tests of the composite were carried out at a crosshead speed of 2 mm/min and a gauge length of 120 mm in accordance with ASTM D3039. The load cell of 100 KN was used for these tests. The composite test samples were conditioned for 24 h at 23 °C temperature and 50% relative humidity. During the tensile test, tension was applied until fracture occurred. It has been well known that the mechanical behavior of composite materials based on a thermosetting matrix and an inorganic reinforcement depends much on the interactions between the constituents of the composite. In general, the addition of flame-retardant particles into polymer composites can cause a reduction in the mechanical properties, as the interfacial adhesion between the fiber and resin matrix is impaired by the addition of fillers.

**Figure 3:**
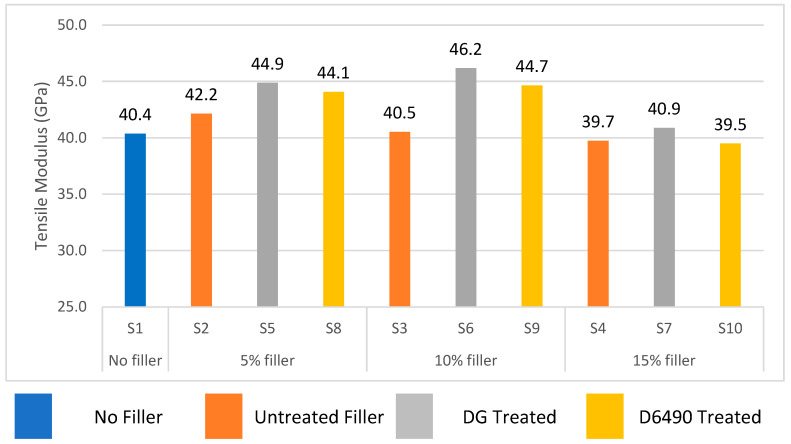
Effect of filler % age and silane treatment on tensile modulus.

The effect of silane treatment on the tensile modulus of a glass epoxy composite with different filler concentrations is given in Figure 3. It can be noticed that the surface modification of aluminum hydroxide particles with silane-coupling agents affects the tensile modulus of the glass fiber-reinforced epoxy composite. The tensile modulus of the epoxy glass composite increases by adding DG and D6490-treated fillers up to 10 wt.%, and a maximum value of tensile modulus was achieved in S6 and S5 with DG-treated 10 wt.% and 5 wt.% aluminum hydroxide particles, respectively. It is very clear from Figure 3 that by increasing particles to 10 wt.%, the tensile modulus was increased, and by increasing particles concentration to 15 wt.%, the tensile modulus was decreased. It may be due to the higher concentration of filler particles which form agglomerates; these agglomerates cause an incomplete mixing of aluminum hydroxide particles in epoxy resin, which creates stress concentration points, consequently decreasing the tensile properties of composite samples.

**Figure 4:**
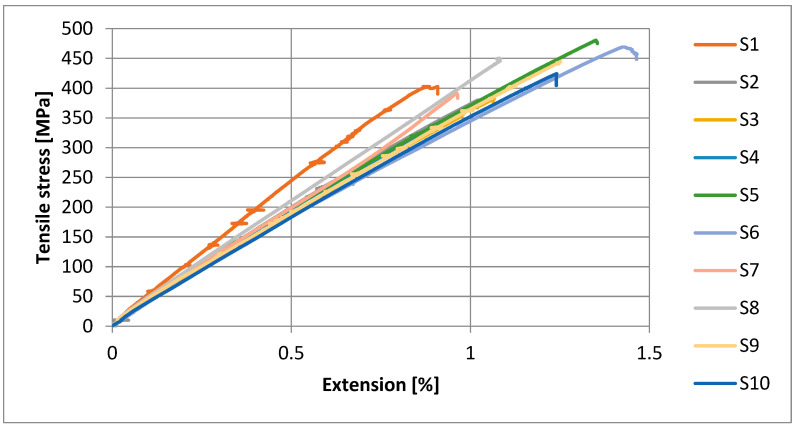
Stress–strain curves of tensile test.

The stress–strain curves of the tested composite samples are shown in Figure 4. It is very fascinating to note that the addition of untreated aluminum hydroxide particles up to 5 wt.% (e.g., Sample S2) as a flame retardant did not have a negative impact on the tensile properties of the composite laminates [31]. But with untreated 10 wt.% and 15 wt.% filler concentration, S3 and S4 presented a decrease in tensile strength. Furthermore, composites samples containing 10 wt.% DG-treated particles (e.g., sample S6) show a massive increase in tensile strength. By introducing 15 wt.% treated filler particles, the tensile strength of the composite reduced due to the agglomeration and excessive amount of filler particles. The addition of 10 wt.% DG-treated particles increase the tensile strength of the epoxy glass composite with respect to the others. From the results, it is observed that the 5 wt.% and 10 wt.% DG-treated filler particles exhibit a higher tensile strength than all the others. The increasing trend of tensile strength is observed by the addition of filler particles up to 10 wt.%, and then it keeps decreasing, which is due to the excess number of particles that form agglomerates in epoxy resin. These agglomerates reduce the interaction between the epoxy, glass, and aluminum hydroxide particles and create stress concentration points, which leads to the decrease in tensile strength of the composite [31]. But the D6490 treatment did not exhibit any significant effect on the tensile strength of the laminates. 

**Figure 5:**
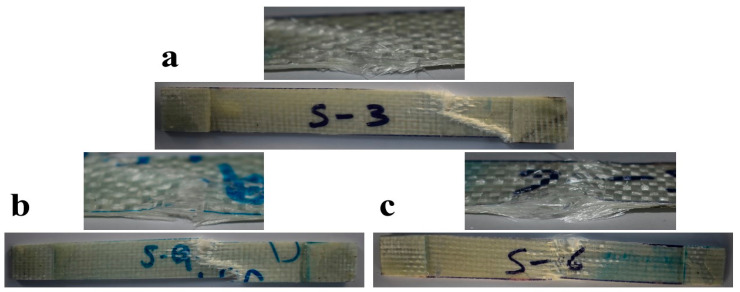
Photographic images of post-tensile strength test samples (**a**) S3 (**b**) S9 (**c**) S6.

The photographic images of fractured surfaces of composites after the elongation test are presented in Figure 5, which indicate the ductile behavior of the composite.

### 3.2. Flexural Properties

Composite samples were conditioned for 24 h at 23 °C temperature and 50% relative humidity before tests. Flexural tests were conducted at a crosshead speed of 2 mm/min. Samples were loaded at three bending points at a span-to-width ratio of 16:1. The flexural test setup is shown in Figure 2b. The flexural strengths and flexural moduli are improved by the addition of aluminum hydroxide particles in the composites. This is because the filler nanoparticles restrict the movement of fabric layers in the composite when the specimen is subjected to a bending load. But at a higher filler content, the effectiveness of this reinforcement is reduced by agglomerated filler particles, improper wetting, and poor interface. Also, increased toughness by the addition of aluminum hydroxide particles to the epoxy matrix contributes to the enhancement of shear properties of the composites [32].

**Figure 6:**
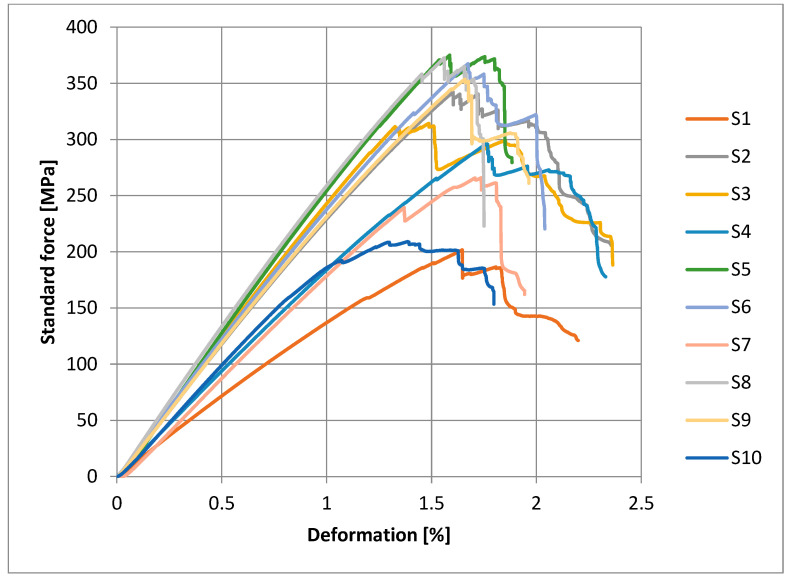
Strength vs. deformation curves of bending test.

**Figure 7:**
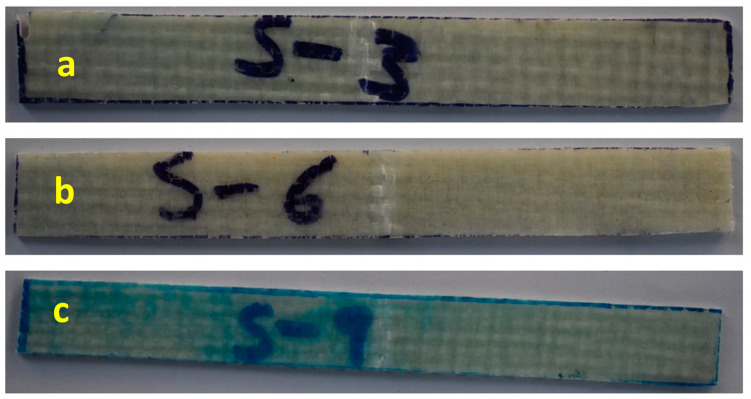
Post-test photographic images of samples of 3-point bending test (**a**) S3 (**b**) S6 (**c)** S9.

Flexural properties represent the resistivity of the materials against bending deflection on the application of energy, and good flexural strength indicates that the materials have brittle properties and high hardness. Figure 6 shows the variation of flexural strength verses the deformation of samples with various filler particles content and treatment. Also, the results indicated that the 5 wt.% and 10 wt.% DG-treated filler particles-loaded composites exhibit ductile behavior (whose images are shown in Figure 7) with excellent flexural strength as compared to the composites without filler particles and 15 wt.% particles. 

In Figure 6, the glass fiber-reinforced epoxy composite results show that the flexural strength increases by adding aluminum hydroxide particles up to 5 wt.%, and a further increase in flexural strength is due to the treatment of aluminum hydroxide particles with DG and D6490, which improves the epoxy glass interfacial bonding. The highest value of flexural load that specimen can withstand was achieved at 10 wt.% DG-treated aluminum hydroxide filler particles. And the addition of 15 wt.% untreated, DG and D6490-treated aluminum hydroxide particles shows a decrease in the flexural strength of laminates. Specimen S6 and S5 with 10 wt.% and 5 wt.% DG-treated aluminum hydroxide particles exhibit superior flexural properties, respectively. This is because the filler nanoparticles restrict the movement of fabric layers when the specimen is subjected to a bending load. But, at a higher filler content (e.g., 15 wt.%), the effectiveness of this reinforcement is reduced by agglomerated particles, improper wetting, and poor interfacial adhesion [33].

### 3.3. Short Beam Shear

**Figure 8:**
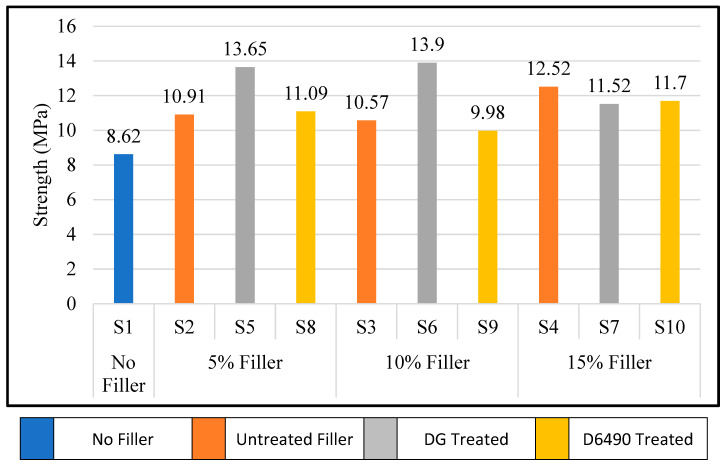
Effect of filler wt.% age and treatment on interlaminar shear strength.

A short beam shear (SBS) test was performed to measure the interlaminar shear strength (ILSS) of the glass fiber-reinforced epoxy composite. The test setup is shown in Figure 2c, where the load was applied at the center of the composite sample, which was located on two roller supports. Composite samples were conditioned for 24 h at 23 °C temperature and 50% relative humidity before tests. Figure 8 shows the effect of filler wt.% age and treatment on the interlaminar shear strength of glass epoxy composites. It is very clear that specimen S2 with untreated 5 wt.% aluminum hydroxide particles shows improved intralaminar shear strength as compared to the specimen S1 without filler particles. The interlaminar shear strength was further enhanced by adding 5 wt.% DG-treated aluminum hydroxide particles in specimen S5. But no significant effect was observed in specimen S8 treated with D6490. Similarly, with 10 wt.% of aluminum hydroxide particles, such as specimen S3 and S9 without silane treatment and with D6490 treatment, respectively, did not show any change in shear strength, but specimen S6 with DG treatment shows an increase in interlaminar shear strength. The 15 wt.% aluminum hydroxide particles (e.g., S4, S7 and S10) did not show any change in interlaminar shear strength (ILSS), which may be due to the incorporation of a large amount of filler particles, which affects the epoxy glass bonding of composites [34].

**Figure 9:**
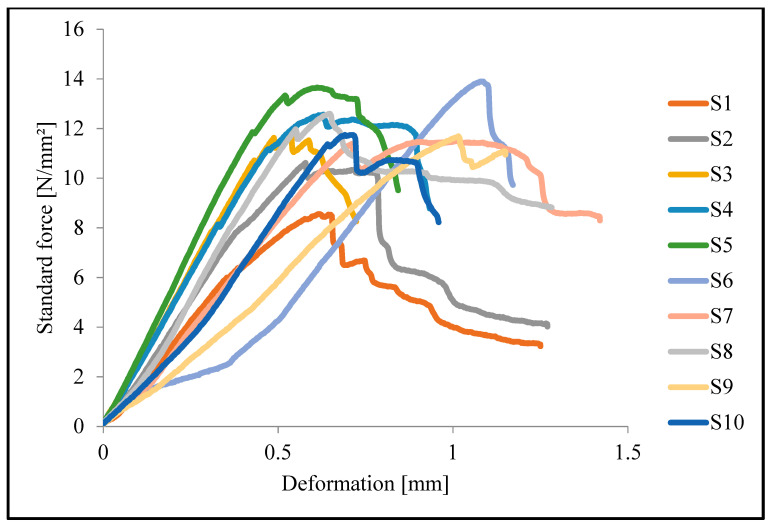
Force vs. deformation curves of short beam shear test.

Our findings in this study are complex, since we are using woven glass fiber-reinforced epoxy composites. Under SBS stress, these materials go through a number of distinct failure and damage patterns until interlaminar shear failure occurs. Several SBS load vs. deformation curves are shown in Figure 9 for different specimens. These curves indicate the increase in load-bearing capacity of epoxy glass composite with DG or D6490-treated aluminum hydroxide particles and with untreated aluminum hydroxide particles. Sample S2 shows the increase in ILSS by adding 5 wt.% aluminum hydroxide particles, and a further increase in strength is shown in S5, which contains 5 wt.% DG-treated aluminum hydroxide particles, and strength is increased by the addition of up to 10 wt.% DG-treated aluminum hydroxide particles, as shown in curve S6. Samples S8 and S9 containing D6490-treated 5 wt.% and 10 wt.% aluminum hydroxide particles, respectively, did not show a relative change in strength. It is indicated from the curves that by increasing flame-retardant particles to 15 wt.% the strength is decreased due to the agglomeration and low distribution of particles in epoxy, which creates the stress concentration point in composites and leads to a decrease in the bonding between the matrix and reinforcement.

## 4. Conclusions

This study investigated the influence of silane-treated aluminum hydroxide on the mechanical performance of glass fiber-reinforced epoxy flame-retardant composites. The aluminum hydroxide fillers help to impart the flame retardancy in the composite materials, but the mechanical performance of the resultant composites is compromised due to compatibility issues with the epoxy matrix. Hence, in this study, we used silane-coupling agents-treated Al(OH)3 filler particles to improve the mechanical performance of glass fiber-reinforced epoxy composites. Two different silane-coupling agents (Dynasylan^®^ 6490 and Dynasylan Glymo) were used to treat the aluminum hydroxide fillers, and the treated fillers were used in different fractions (5%, 10% and 15%). Both the silane-coupling agents fraction used for treatment and the fillers fraction added to the composites were varied to determine the most optimum combination. The Dynasylan Glymo-treated fillers showed better performance in terms of tensile strength, flexural strength and short beam shear strength as compared to the D6490-treated aluminum hydroxide particles. We found that the filler concentration also has a significant effect on the mechanical performance of the treated samples. By increasing the filler concentration from 5 wt.% to 10 wt.%, the mechanical performance of the treated samples also increased. The developed glass fiber-reinforced fire-retardant composites have potential applications in luggage bags as an alternative material to fabric, metal and plastics, with improved mechanical performance, durability and fire protection. The lightweight luggage bags will also contribute toward less carbon emissions in the environment. 

## Figures and Tables

**Table 1:** List of materials with their specifications.

Sr. #	Materials	Specifications	Supplier
1	Epoxy	Epoxy based on bisphenol-A epichlorohydrin	Aditya Birla Chemicals (Thailand)
2	Chemical Resistant Curing Agent	Isophorone diamine (IPDA)	Aditya Birla Chemicals (Thailand)
3	Reinforcement	Plain woven glass fabric, Areal density = 198 ± 4	China
4	Aluminum Hydroxide Al (OH)_3_	Extra pure grade	Sigma Aldrich
5	Dynasylan^®^ Glymo	Lab-grade 99% pure	EVONIK industries
6	Dynasylan^®^ 6490	Lab-grade 99% pure	EVONIK industries

**Table 2:** List of composite samples.

Sr. #	Sample ID	Matrix	Fillers	Treatment	Filler %
1	S1	CR epoxy	No	No	0
2	S2	CR epoxy	Al (OH)_3_	No	5
3	S3	CR epoxy	Al (OH)_3_	No	10
4	S4	CR epoxy	Al (OH)_3_	No	15
5	S5	CR epoxy	Al (OH)_3_	DG	5
6	S6	CR epoxy	Al (OH)_3_	DG	10
7	S7	CR epoxy	Al (OH)_3_	DG	15
8	S8	CR epoxy	Al (OH)_3_	D6490	5
9	S9	CR epoxy	Al (OH)_3_	D6490	10
10	S10	CR epoxy	Al (OH)_3_	D6490	15

D6490 = Dynasylan^®^ 6490 treated, DG = Dynasylan^®^ Glymo Treated.

**Table 3:** List of equipment with their test standards and purposes.

Sr. #	Equipment	Method	Standard
1	Universal testing machine (UTM)	Tensile properties Flexural properties Short beam shear	ASTM D3039-17 ASTM D790-17 ASTM D2344-22

## Data Availability

Not applicable.
